# Spectrum of HRCT findings among asymptomatic and recovered COVID-19 patients: how did they impact the clinical decision?

**DOI:** 10.1186/s43055-020-00356-2

**Published:** 2020-12-01

**Authors:** Ahmed Samir, Mohamed Saied Abdelgawad, Ayman Ibrahim Baess, Hebatallah Hassan Mamdouh Hassan

**Affiliations:** 1 Department of Radiodiagnosis and Intervention, Faculty of Medicine, University of Alexandria, 17 Champollion Street, El-Messalah, Al-Kartoom Square, Al-Azareta, Alexandria, 21526 Egypt; 2 Department of Radiodiagnosis and Intervention, National Liver Institute, University of Menoufia, Shibin El Kom, Egypt; 3 Department of Chest Diseases, Faculty of Medicine, University of Alexandria, Alexandria, Egypt

**Keywords:** COVID-19, Asymptomatic, HRCT, Clinical, Decision

## Abstract

**Background:**

Decisions about asymptomatic COVID-19 patients are always critical, either during initial screening or during recovery. Spread of infection will be inevitable if those patients were left non-isolated. This study aimed not only to survey spectrum of HRCT findings of COVID-19 among asymptomatic and recovered patients but also to record unexpected results and document their impact upon the clinical decision.

**Results:**

The study was retrospectively conducted, during June and July 2020, on 120 patients proved with COVID-19, during initial HRCT screening or delayed following announcement of recovery. All patients were completely asymptomatic. They included 72 males and 48 females (60%:40%). Their age ranged from 10 to 58 years (mean 35.95 ± 12.25 SD). HRCT was analyzed by three expert consultant radiologists in consensus. Among asymptomatic initially screened COVID-19 patients, additional to GGOs, bilateral consolidative changes were unexpectedly found together with secondary fibrosis (23.3% and 10%). HRCT results significantly impacted the clinical decision (*P* < 0.0001); PCR had to be repeated with home isolation (43.3%). Infected health care providers had to stop their duty immediately (20%). Isolated hospitalization replaced routine ward admission (25%). Cautious surgical interference was performed using full personal protective equipment (PPE) (8.3%). Among asymptomatic recovered COVID-19 patients, unexpected large lesions (> 3 cm) were found (70%). Near 50% of lung volume was persistently affected (10%). Secondary fibrosis was striking (33%). Encysted hydro-pneumothorax persisted for a whole month (1.7%). “No-isolation” decision remained unchanged because of clinical and laboratory stability; however, steroids were prescribed to speed lung recovery.

**Conclusion:**

HRCT findings among asymptomatic and recovered COVID-19 patients can be unexpected and can definitely impact the clinical decision.

## Background

COVID-19 is a highly infectious disease that started in Wuhan in December 2019 and rapidly spread all over the world to be announced as a pandemic by the WHO in March 2020. Infection can be transmitted through respiratory air droplets or via direct contact with contaminated surfaces [1–4].

While COVID-19 is mainly manifested by fever, cough, dyspnea, and chest tightness, it was reported that around 1-5% of patients can be asymptomatic. Those asymptomatic patients are recommended to be isolated to avoid further spread of infection [5–7]. Patients are announced to be recovered from the disease and discharged from the hospital when they have two consecutive negative PCR swab tests at least 24 h apart. Those patients are mostly asymptomatic [8].

Decisions about asymptomatic and recovered patients are always critical. They need to be highly accurate and also rapid because infection spread will be inevitable if those patients were left non-isolated. Considering the PCR low sensitivity, time consumption, high cost as well as non-availability in some countries, HRCT screening has expanded to involve not only those persons who had contact with proved COVID-19 patients but also every patient who will be admitted to a health facility to receive any kind of medical care. This study aims to record unexpected HRCT findings among asymptomatic and recovered COVID-19 patients, also to evaluate their impact upon clinical decision.

## Methods

### Study population and medical records review

This study was retrospectively conducted, during June and July 2020, on 120 patients proved with COVID-19; 60 patients were discovered during initial HRCT screening while other 60 patients had delayed abnormal CT findings for 2 to 4 weeks following announcement of recovery. All patients were completely asymptomatic. They included 72 males and 48 females (60%:40%). Their age ranged from 10 to 58 years (mean age was 35.95 ± 12.25 SD).

The study was approved by The Ethics Committee of our University Hospital. Patient consent was waived by the Research Ethics Board with assurance of respect of confidentiality of the patients and medical records.

Inclusion criteria were completely asymptomatic patients with positive HRCT results during one of the following scenarios: (1) Initial HRCT screening for asymptomatic patients with recent contact history with proved COVID-19 patients or asymptomatic patient with other irrelevant medical condition necessitating hospital admission (both proved positive for COVID-19 using PCR swab tests earlier or later). (2) Follow-up CT scans for asymptomatic recovered COVID-19 patients, performed 2-4 weeks after two consecutive negative PCR results announcing patient recovery.

Exclusion criteria were (1) degraded CT scans quality because of respiratory motion artifacts. (2) Any chest symptom relevant to COVID-19.

Evaluation of the impact of HRCT results on the clinical decision was done by a single consultant pulmonologist who has 19 years’ experience in the field of infectious lung diseases. Additional correlation with the oxygen saturation and laboratory tests among the recovered patients was also performed.

### CT scanning and parameters

Two MDCT machines were used: Siemens SOMATOM Sensation 64 (Germany) and Toshiba Aquilion CXL/CX 128 (USA). The following CT scanning parameters were used: 1 mm slice thickness, 1 mm detector collimation, 0.6-0.9 s tube rotation, helical mode volumetric HRCT with 100-120 kVp and 80-200 mA, according to the weight of patients and clinical indication. Intra-venous contrast administration was not used.

### CT analysis

CT scans were evaluated in consensus by three consultant radiologists who were informed with the clinical data and have 15, 19, and 25 years of experience in chest imaging. Multi-planner reconstruction (MPR) was used for image analysis. Post-processing maximum intensity projection (MIP) and minimum intensity projection (Min-IP) reconstructions were performed. Each CT scan was evaluated according to (1) site of lung involvement, (2) universally agreed CT findings with COVID-19 including ground-glass opacities (GGO) with or without consolidative changes in addition to special signs such as “Atoll sign” and “Crazy paving pattern” [3], manifestations of bronchial or pleural involvement.

### Statistical analysis

The prevalence rate of HRCT findings was estimated as the percentage of patients showing any criterion. Data were compared using a chi-square test and *P value* < 0.05 was considered statistically significant. Online calculators were used (https://www.socscistatistics.com/).

## Results

Wide spectrum of HRCT findings were found in this study among both asymptomatic initially screened patients and asymptomatic recovered patients. They included unexpected results regarding the number, the size of the lesions, the site and extension of lung involvement, and the different morphological CT features. All HRCT findings are detailed in Table 1.

**Table 1:** Distribution of patients according to “Prevalence of HRCT findings”

Prevalence of HRCT findings	Asymptomatic COVID-19 patients			
	Initially screened		Recovered	
	***N*** (60)	%	***N*** (60)	%
* Unilateral versus bilateral lung involvement				
Unilateral (one lung involved)	18	30%	Not detected	
Bilateral (both lungs involved)	*42*	*70%*	*60*	*100%*
* Size and extension of the lesions				
Less than 3 cm	*42*	*70%*	10	16.7%
Peripheral (> 3 cm longest dimension but < 3 cm extension from pleural surface).	15	25%	*42*	*70%*
Peripheral (> 3 cm extension from pleural surface but < 50% of lobar involvement) or peri-bronchial (> 3 cm)	3	5%	6	10%
Diffuse lobular pattern (extending proximally > 3 cm from pleural surface and > 50% of lobar involvement)	Not detected		2	3.3%
* Number of the lesions (excluding diffuse lobar pattern)				
Less than 3	24	40%	Not found	
More than 3	*36*	*60%*	60	*100%*
* HRCT findings				
**GGO nodules or patches**	***46***	***76.7%***	**36**	**60%**
“Atoll sign” (*starting organization*)	6	10%	24	40%
“Air bubble sign”	1	1.7%	4	6.7%
*Secondary fibrosis*	6	10%	20	33.3%
“Crazy paving pattern”	6	10%	Not found	
**GGOs mixed with consolidative changes**	**14**	**23.3%**	**24**	**40%**
“Curvilinear” *consolidations or fibro-consolidations*	2	3.3%	24	40%
* Associated signs/findings				
Hydro-pneumothorax	Not detected		1	1.7%
Bronchial wall thickening and traction bronchiectasis	Not detected		1	1.7%

* Highest values are demonestrated in italic

### Regarding the asymptomatic patients with positive initial HRCT screening

#### PCR results and clinical course

Timing of positive PCR results was variable; only 31/60 patients (51.7%) had positive PCR swab results at the first trial while 17 patients (28.3%) had positive results at the second trial and the remaining 12 patients (20%) had late positive results at the third trial.

Forty-two patients had their initial PCR swab tests before HRCT screening; 26 patients among them had negative PCR results, and then proved to be positive later (2-4 days) after HRCT screening. Other three patients, who were admitted for non-pulmonary medical conditions and performed HRCT screening, showed negative first PCR results then proved to be positive later.

All patients were persistently asymptomatic till the PCR proof of COVID-19 infection.

#### HRCT findings

**Fig. 1:**
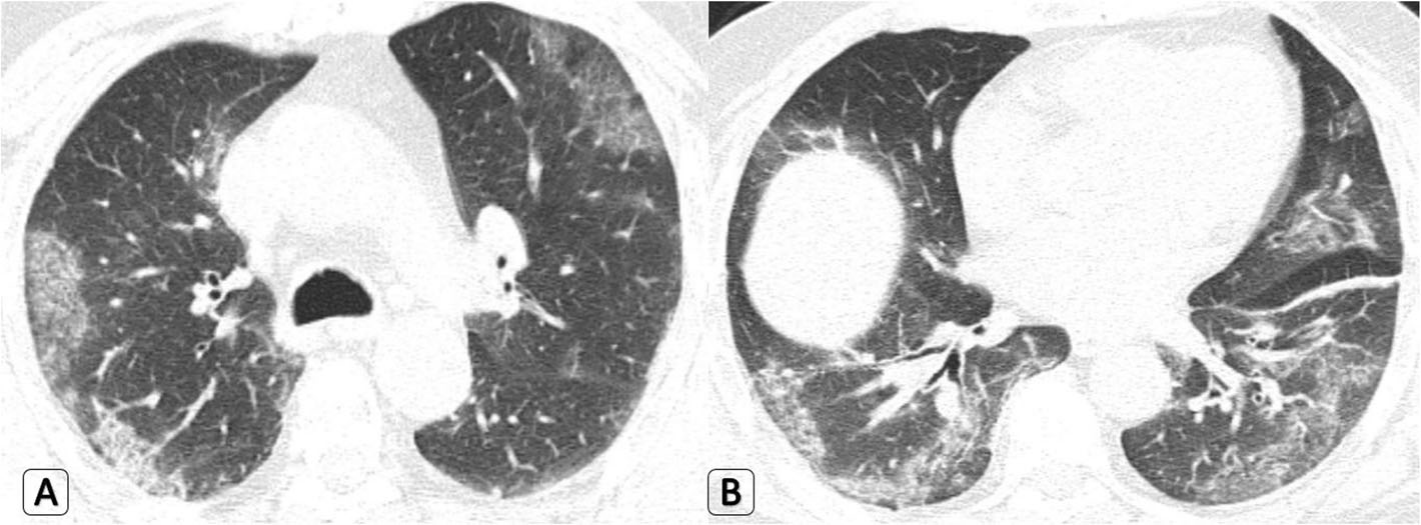
A 45-year-old male patient who had positive contact history with recently proved COVID-19 patient and was completely asymptomatic. **a**-**b** Axial HRCT chest lung window showed bilateral large peripheral located ground-glass patches with mild septal thickening (evolving crazy paving pattern) and left basal fine atelectatic band. Clinical decision was home isolation, medical treatment, and PCR testing. Positive PCR result for COVID-19 was proved later

Bilateral lung involvement was predominant in 42/60 (70% of patients), the number of lesions exceeded three in 36/60 (60% of patients) and the size of lesions exceeded 3 cm in 18/60 (30% of patients) (Fig. 1).

**Fig. 2:**
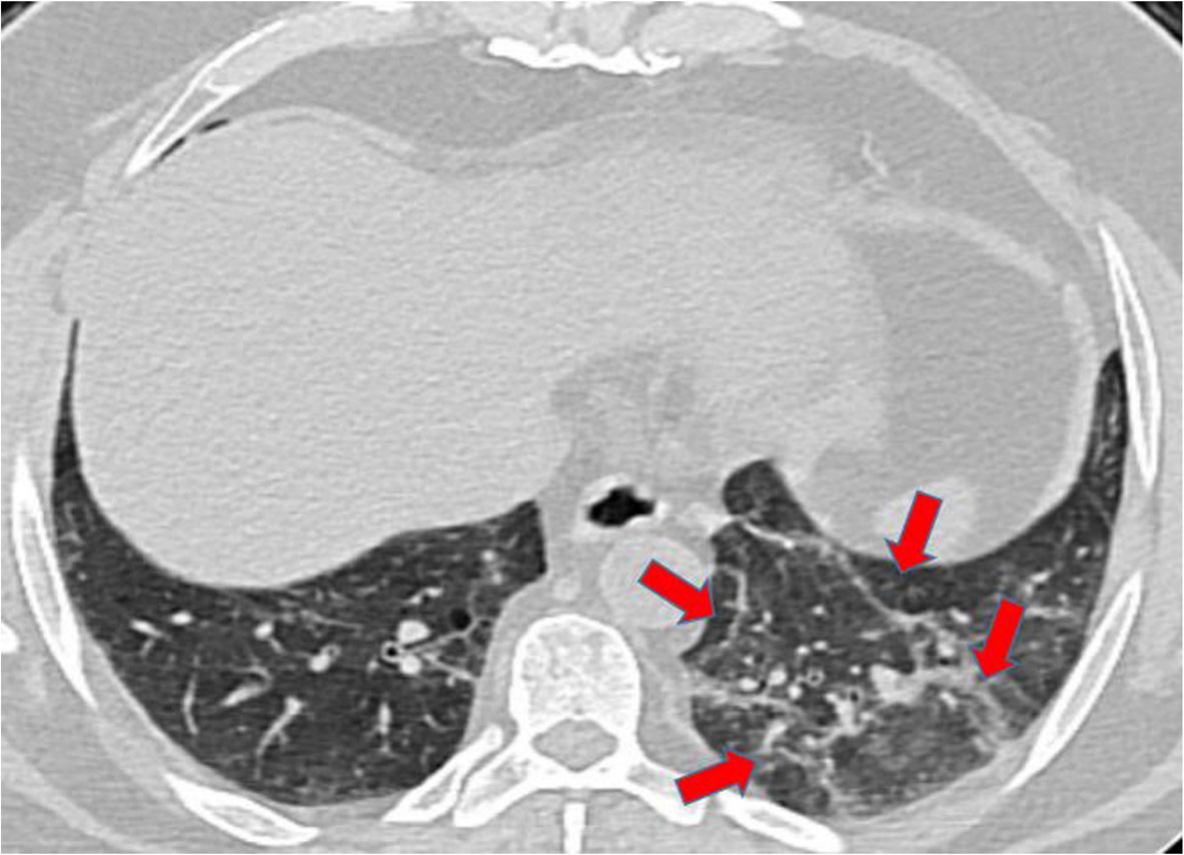
A 52-year-old male patient who had positive contact history with recently proved COVID-19 patient and was completely asymptomatic: Axial HRCT chest lung window showed left lower lobar patchy GGOs with secondary fibrosing changes (red arrows). Clinical decision was home isolation, medical treatment, and PCR testing. Positive PCR result for COVID-19 was proved later

In addition to GGOs, consolidative changes were found in 18/60 (23.3% of patients). Atoll sign that denotes organization process of the disease and even secondary fibrosing changes were also found (each in 10% of patients) (Fig. 2). Even the curvilinear bands with sub-pleural sparing, that denote healing process, were found in 3.3% of patients.

#### Impact of HRCT findings on clinical decision

The abovementioned HRCT results obviously impacted the clinical decision, as detailed in Table 2. Modified or new clinical decision was obvious in 44/60 patients (73.3%). Significant relation between HRCT positive findings among asymptomatic initial screened COVID-19 patients and clinical decision for these patients was statistically proven in Table 3 with *P* value < 0.00001*.*

**Table 2:** Classification of the included patients according to clinical situation and impact on clinical decision after HRCT results

Group of patients:	Included patients:		No	%	Clinical decision changes:
**Asymptomatic** **(Initially screened)**	**[1]**	**People** with positive contact history with proved COVID-19 patients:	30/60 patients (including): - First degree relatives (17). - Work colleagues (13). * N.B: 19/30 patients (63%) had negative prior first PCR results.	*50%* 28.4% 21.6% 31.7%	Home isolation, medical treatment and PCR testing instead for uncontrolled release. (Repeating PCR for those 19 patients who showed initial negative prior PCR swab tests’ results → Proved positive later).
	**[2]**	**Health care providers** with positive contact history with proved COVID-19 patients**:**	12/60 patients (including): - 7 doctors - 5 nurses. * N.B: 7/12 patients (58.3%) had negative prior first PCR results.	*20%* 11.7% 8.3% 11.7%	Home isolation, medical treatment and PCR testing instead of continuing providing health service. (Repeating PCR for those 7 patients who showed negative prior PCR swab tests’ results → Proved positive later).
	**[3]**	**Dialysis unit:** Chest HCRT screening before admission for dialysis.	4/60 patients known with end stage renal disease (ESRD)	*6.7%*	PCR testing, isolated hospitalization and isolated dialysis.
	**[4]**	**Oncology unit:** Chest HRCT screening before admission for receiving chemotherapy.	2/60 patients (including): - Metastasizing colon cancer. - Breast cancer.	*3.3%*	PCR testing, isolated hospitalization and delay of chemotherapy cycles.
	**[5]**	**Emergency unit:** Chest HRCT screening before surgery planning.	2/60 patients (including): - RTA. - Abdominal exploration.	*3.3%*	* PCR testing and isolated hospitalization with for the patients. * Full PPE order for the surgery stuff.
	**[6]**	**Intervention unit:** Chest HRCT screening before interventional procedure.	1/60 patient asked for CT guided biopsy	*1.7%*	* PCR testing, isolated hospitalization, and delaying biopsy appointment.
	**[7]**	**Radiology unit:** Chest HRCT screening during or before performing extra-thoracic radiological investigation.	9/60 patients (including): - CT enterocolonography (5). - Urgent CT abdomen (3). - Dorsal spine MRI as metastatic workup for cancer prostate (1).	*15%* 8.3% 5% 1.7%	* Strict infection control measures for CT and MRI machines. * PCR testing then home isolation and isolated hospitalization for 3 and 6 patients respectively. * Full PPE instructions for the surgery stuff required for the three urgent cases.
**Asymptomatic (Recovered)**	Follow up CT after two consecutive negative PCR results for previous proved COVID-19 patients.		60 Patients * All had unremarkable laboratory tests and normal O_2_ sat levels.	*100%*	* Whatever the unexpected CT findings or lesions size, clinical decision remain unchanged; no need for re-isolation or re-hospitalization. * Steroids were prescribed to speed lung recovery. * Long term follow up for lung fibrosis was advised.

**Table 3:** Statistical analysis of significance of HRCT positive findings among asymptomatic initially screened COVID-19 patients on clinical decision

Positive HRCT initially screened patients	Clinical decision changed	Clinical decision unchanged	Total
Initial (1st) PCR positive	15 (22.7)	16 (8.3)	**31**
Initial (1st) PCR negative	29 (21.3)	0 (7.7)	**29**
**Total**	**44**	**16**	**60**

*Chi-square value (20.4) and *P* value (< 0.00001). As *P* value (< 0.05) is considered significant → so there is significant relation between HRCT positive findings among asymptomatic initial screened COVID-19 patients and clinical decision for these patients

* This footnote is the consequent value and interpretation of the given values in above-mentioned table

**Fig. 3:**
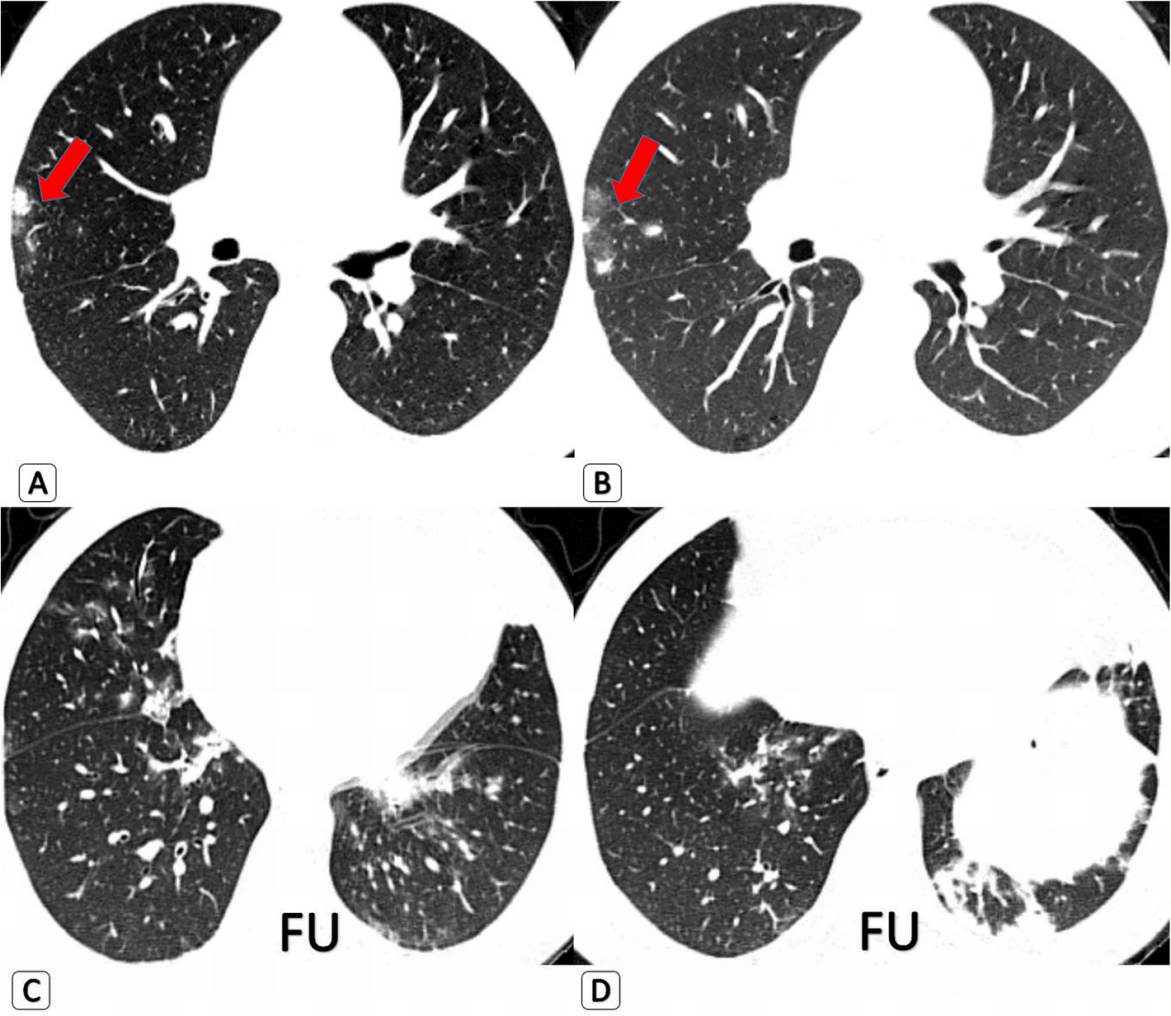
A 33-year-male patient with SLE and ESRD who was completely asymptomatic however underwent screening HRCT scan prior to hospital admission for dialysis. **a** and **b** Axial HRCT of chest (lung window) shows initial presentation by unilateral right upper peripheral sub-pleural four solid nodules with GG “halo sign” (red arrows). They were conflicted as either COVID-19-related or SLE-related nodules. Isolated hospitalization, PCR testing and special isolated unit dialysis was the clinical decision. **c** and **d** Follow-up study for the patient 3 days later, he was also still completely asymptomatic: Axial HRCT chest showed de novo multiple bilateral GGO patches and left basal linear consolidation. PCR test for COVID19 was carried out and proved positive

Home isolation and medical treatment were the modified clinical decision, instead of uncontrolled release, for 26/42 patients (62%) who had negative PCR swab tests’ results before HRCT. Repeating PCR tests was requested for them and PCR proved positive 2-4 days later. Among those 42 patients, 12 patients were health care providers and had to stop their duty immediately. Home isolation or isolated hospitalization instead of routine ward admission was the modified clinical decision for three and 15 patients respectively (Fig. 3).

**Fig. 4:**
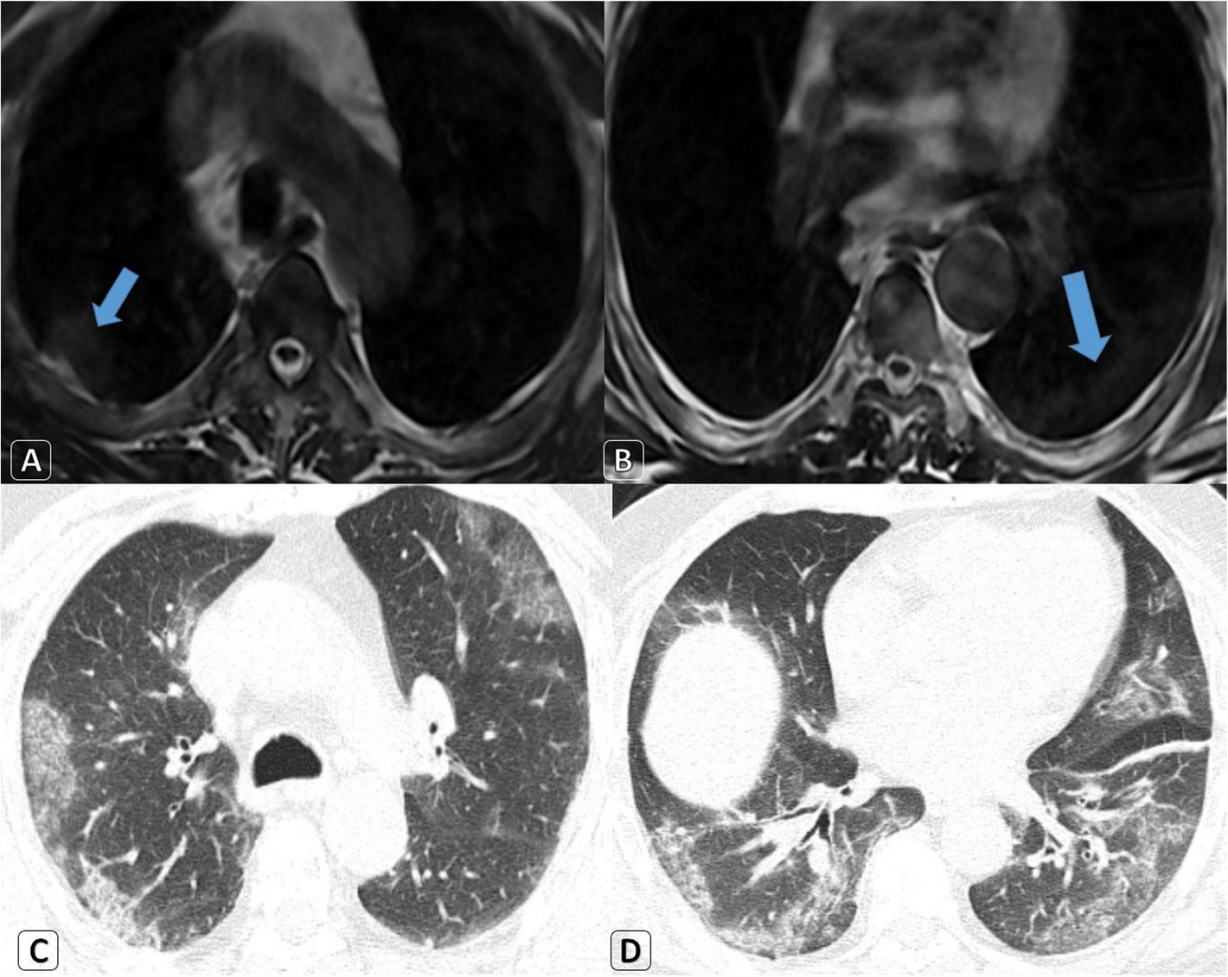
A 58-year-old male patient with history of prostatic cancer. He had a visit to the radiology unit to do a whole spine MRI because of back pain. **a**-**b** Dorsal spine MRI using high standard infection control measures revealed: axial T2WI showed the same patches with minimal overlying pleural reaction (blue arrows). **c**-**d** Screening axial chest HRCT lung window revealed bilateral large peripheral located ground-glass opacities mixed with consolidative changes. Clinical decision was isolated hospitalization, medical treatment, and PCR testing. Positive PCR result for COVID-19 was proved later

Cautious surgical interference using full personal protective equipment (PPE) was carried out in five urgent surgeries instead of routine infection control measures. Delaying a scheduled chemotherapy cycle was decided for two patients. Delaying an elective interventional procedure was also decided for one patient. Strict infection control measures were requested for the radiology unit, which was visited by nine COVID-19 patients (Fig. 4).

### Regarding the asymptomatic recovered patients

#### Residual HRCT findings (2-4 weeks after announcement of patient recovery by two consecutive negative PCR swab tests)

**Fig. 5:**
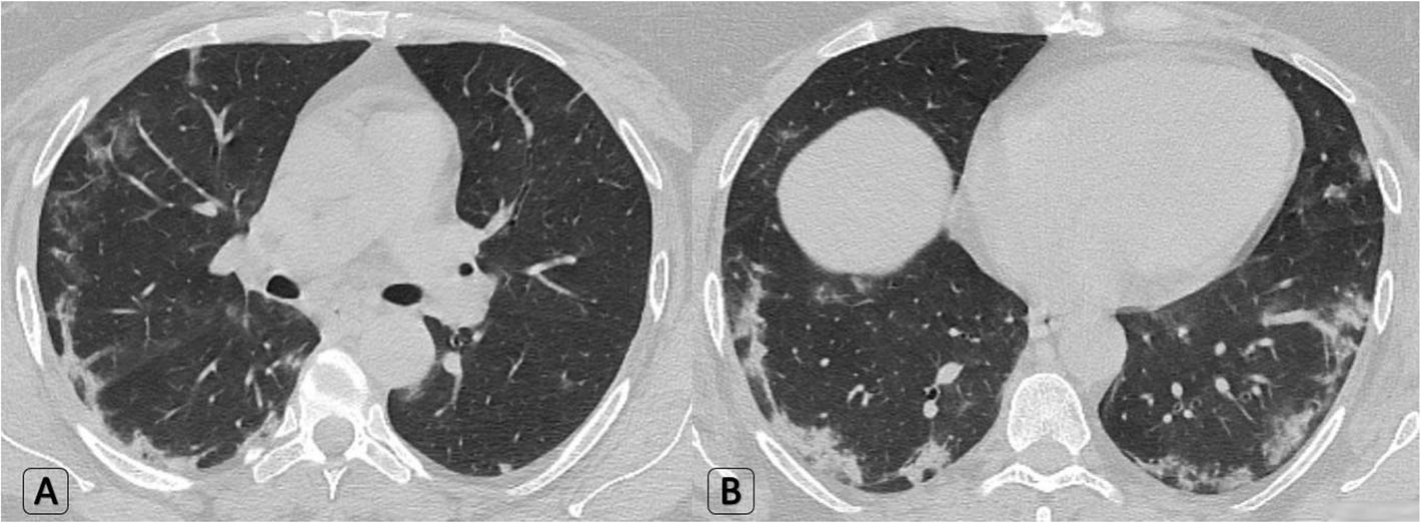
A 42-year-old male patient known for COVID-19: Follow-up HRCT scans performed 2 weeks after announcing recovery. Patient was completely asymptomatic. O_2_ saturation = 97%. Unremarkable laboratory results. **a**-**b** Axial HRCT lung window showed residual bilateral sub-pleural curvilinear fibro-consolidative patches. Clinical decision was no need for re-isolation or re-hospitalization

**Fig. 6:**
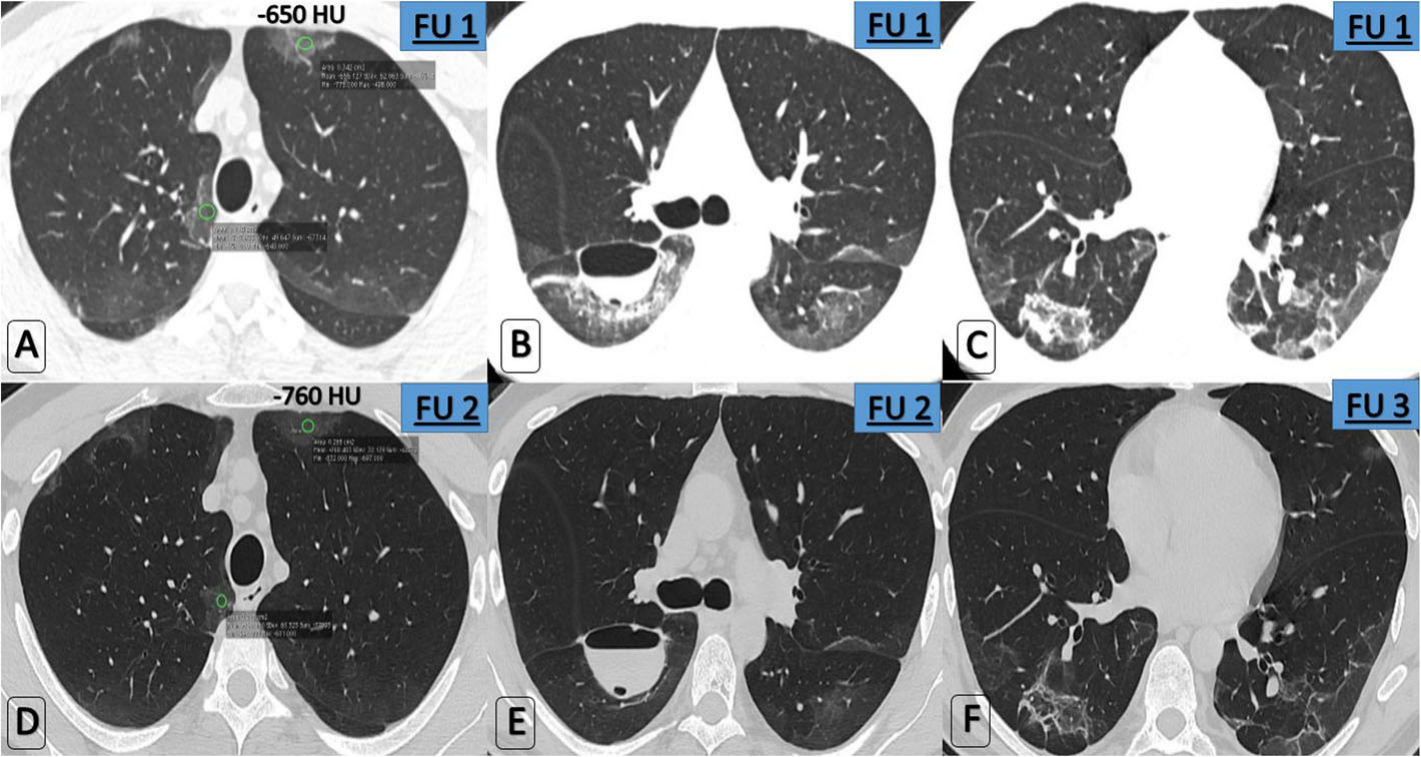
Serial follow-up for a completely asymptomatic recovered 44-year-male patient proved with COVID-19. **a**, **b**, **c** First follow-up study edited 4 days after two consecutive negative tests’ results announcing recovery. Axial HRCT chest—lung window; (**a**) higher GG attenuation (−650 HU), (**b**) process was complicated by encysted fissural hydro-pneumothorax surrounded by dense GGO and consolidative changes, (**c**) Peripheral dense organization is noted “Atoll sign.” **d**, **e**, **f** Next follow-up study edited 24 days later with axial HRCT chest—lung window; (**d**) GGO is approximating normal lung parenchyma (−760 HU), (**e**) Near-total resolution of the dense GGO and consolidations surrounding the encysted fissural collection which retained the same size, however, noticed increased fluid component and decreased internal air, (**f**) disappearance of the “Atoll sign” and lower GGO values. Laboratory tests were unremarkable and O_2_ saturation in room air was 96%. Clinical decision remains unchanged: No need for re-isolation or re-hospitalization

Expected simple GGOs were found only in 12/60 (20% of patients) while 40% of patients showed unexpected persistent curvilinear fibro-consolidative changes and also fibrosis on top of GGOs was found in 20/60 (33.3% of patients) (Fig. 5). One patient had persistent encysted hydro-pneumo-thorax for a whole month after announcing recovery (Fig. 6). One patient expressed traction bronchiectatic changes sequel to the lung fibrosis.

**Fig. 7:**
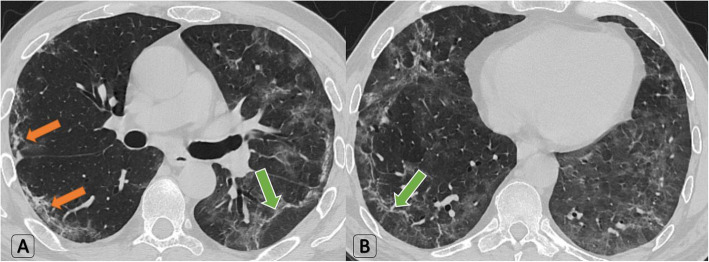
A 37-year-old male patient known for COVID-19: Follow-up HRCT scans performed 18 days after announcing recovery. Patient is completely asymptomatic. O_2_ saturation = 95%. Unremarkable laboratory results. **a**-**b** Axial HRCT lung window showed residual bilateral large GGOs (approximating 50% of lobar volume), atoll sign (green arrows), and curvilinear fibrotic bands (orange arrows). Clinical decision was no need for re-isolation or re-hospitalization

Small-sized residual lesions (less than 3 cm) were only found in 10/60 (16.7% of patients) while unexpected large lesions (> 3 cm) were found in 70% of patients. Moreover, near 50% of lung involvement was found in 10% of patients (Fig. 7).

#### Impact of HRCT findings on clinical decision

All recovered patients in this study had unremarkable laboratory tests and normal range of O_2_ saturation (95-100% O_2_ Sat/RA), even those patients with secondary fibrotic changes (95-96% O_2_ Sat/RA). Consequently, the clinical decision remained unchanged with no need for re-isolation or re-hospitalization whatever the prolonged and even unexpected residual CT findings. However, steroids were prescribed for all patients. Also, long term follow-up was recommended to those patients who had persistent secondary fibrosing changes to evaluate its effect on lung function and rule out the possibility for developing secondary interstitial lung fibrosis.

## Discussion

This study surveyed the different HRCT findings among the asymptomatic and recovered patients proved for COVID-19, also recorded the unexpected results, and documented their impact on the clinical decision.

### Regarding the asymptomatic patients with positive initial HRCT screening

Bilateral lung involvement was predominant in this study (70%) conversely to Meng H et al. [9] who found unilateral lesions predominant in 58.6% of their patient, still the current study agreed with them regarding the prevalence of multiple lesions (more than 3) in 60% of patients. Consolidative changes mixed with GGOs were strikingly more than that noticed by Meng H et al. [9] (23.3% compared to 5.2%) and unexpectedly disagreeing with Hu Z et al. [10], Youssef I et al. [11], and Chang MC et al. [12] who found only pure GGOs (100%) without any consolidative changes. “Crazy paving pattern” was depicted in 10% of patients, similar to Meng H et al. [9]. This may be explained by a higher viral load or different viral strains yielding unexpected more pathology despite being asymptomatic. Further researches may be needed for explanation of this notice.

These positive HRCT findings in asymptomatic initially screened patients had a significant impact on the clinical decision as statistically proved (*P* value < 0.0001) especially among those 29 patients who had initial prior negative PCR swab tests. Modified or new clinical decisions have been made as previously detailed in Table 2 in order to contain infection spread and to protect other patients and health care providers.

Based on the variable timing of positive PCR results among current asymptomatic initially screened patients, this study agreed with Ai T et al. [13] that CT is a better tool than PCR in detection of COVID-19.

### Regarding the asymptomatic recovered patients

During COVID-19 disease recovery and healing process, the consolidative changes in the current study were less than that found in Pan F et al. [14] (40% compared to 75%). Gradual disappearance of these consolidative lesions and replacement by GGOs was the main follow-up sequel in the current study. These GGOs furtherly dropped in HU attenuation till approximating normal lung parenchymal attenuation. This matches the four-step pathway for COVID-19 pneumonia recovery that was described by Pan F et al. [14]; however, in the current study, secondary fibrosis on top of GGOs was strikingly depicted in 33.3% of patients and even one patient expressed secondary bronchiectatic changes on top. This was not depicted at all by Pan F et al. [14]. The persistence of fibrosis was weird and concerned delayed persistent lung impact. Further long-term researches are advised for this notice.

Agreeing with Sun R et al. [15] who stated that pneumothorax could be one of COVID-19 infection outcomes, one patient showed persistent encysted fissural hydro-pneumothorax for a whole month after announcing recovery.

These positive HRCT findings in asymptomatic recovered patients did not change the current clinical decision whatever their grade or delayed persistence. No need for re-isolation or re-hospitalization was kept as the same decision in all recovered patients. This was explained by the fact of “radiological lag,” which was previously stated for pneumonia recovery by Bruns AH et al. [16] and described that clinical recovery always precedes radiological recovery. This was also enforced by the unremarkable laboratory tests and normal O_2_ saturation levels that were observed among these recovered patients. Steroids were prescribed for these patients to speed lung recovery. Long-term follow-up was recommended also to those patients with persistent secondary fibrosing changes to rule out permanent impact on lung functions or possibility for developing secondary interstitial lung fibrosis.

This study has the advantage for tracing the impact on clinical decision not only surveying CT features but also prolonged follow-up for recovered patient; however, it is limited by the small number of patients and lack of further knowledge about the further clinical outcome for those asymptomatic patients after the end of the isolation period.

## Conclusion

HRCT findings among asymptomatic and recovered COVID-19 patients can be unexpected and can definitely impact the clinical decision.

## Data Availability

The datasets used and/or analyzed during the current study are available from the corresponding author on reasonable request.

## Abbreviations

COVID 19: Coronavirus disease 2019
GGOs: Ground-glass opacities
HRCT: High-resolution computed tomography
RT-PCR: Reverse transcription-polymerase chain reaction
